# Supplementary material for: Attitude towards Intranasal Vaccines and Psychological Determinants: Effects on the General Population in Northern Italy
Source: Vaccines (Basel). 2023 Jan 7;11(1):138. doi: 10.3390/vaccines11010138 (PMC9863592; doi:10.3390/vaccines11010138)
Supplement: Supplementary file 1 [file vaccines-11-00138-s001.zip › vaccines-2127189-supplementary.pdf]

SUPPLEMENTARY MATERIALS

Table S1. Survey Tool

ITEMS	RESPONSE
**Socio-demographic Data**	
Age	(Number)
Education	o Primary school<br>o Secondary school<br>o High school<br>o University (Undergrade or Postgrade)<br>o Post-university (e.g., PhD)
Employment status	o Working<br>o Unemployed<br>o Retired<br>o Student<br>o Occasional worker
Geographical area of origin	o North Italy<br>o Central Italy<br>o South Italy<br>o Italian Islands
Do you work as health care professional?	o Yes<br>o No
Do you suffer from one or more organic diseases? Select one or more of the following options:	o No<br>o Yes, diseases of the respiratory system<br>o Yes, diseases of the immune system<br>o Yes, oncological diseases<br>o Yes, metabolic diseases<br>o Yes, liver diseases<br>o Yes, kidney diseases<br>o Yes, cardiovascular diseases<br>o Yes, psychiatric diseases<br>o Other
**PERCEPTION OF VACCINES**	
1. I have had at least one of the mandatory childhood vaccinations.	o YES<br>o NO
2. I have had non-mandatory vaccines at least once.	o YES<br>o NO
3. In general, I think vaccines are useful.	1-4 (1=Not at all; 4=Very much)
4. In general, I think vaccines can be risky.	1-4 (1=Not at all; 4=Very much)
5. Why do you think vaccines may be a risk? You can select more than one option.	o I do not think vaccines are a risk at all<br>o Personal negative experience<br>o Negative experience of family and/or friends<br>o Negative reported experience of acquaintances<br>o Medical opinions<br>o Media (TV, Internet, etc.), press<br>o Scientific journals
6. Vaccines worry me because of their possible short term side effects.	1-4 (1=Not at all; 4=Very much)
7. Vaccines worry me because of their possible long term side effects.	1-4 (1=Not at all; 4=Very much)

8. In general, I think vaccines can protect myself and my family members from serious diseases.	1-4 (1=Not at all; 4=Very much)
9. In general, I think I am sufficiently informed about vaccines.	1-4 (1=Not at all; 4=Very much)
10. What image/word/phrase comes to mind when you think of vaccines?	(Free answer)
**MODE OF VACCINE ADMINISTRATION**	
11. Generally, the opportunity to choose between intramuscular (needle) and intranasal (spray) route of administration could encourage me to get vaccinations.	1-4 (1=Not at all; 4=Very much)
12. I worry less about nasal vaccine, becauseI consider it less invasive for the immune system and for my health.	1-4 (1=Not at all; 4=Very much)
13. I worry less about nasal vaccine, because it is needle-free.	1-4 (1=Not at all; 4=Very much)
14. I would worry less if my family members received nasal vaccine, because I consider it less invasive for the immune system and for their health.	1-4 (1=Not at all; 4=Very much)
15. I would worry less if my family members received nasal vaccine, because it is needle-free.	1-4 (1=Not at all; 4=Very much)
16. I think intranasal vaccine may cause fewer side effects.	1-4 (1=Not at all; 4=Very much)
17. I think intranasal vaccine has adequate efficacy.	1-4 (1=Not at all; 4=Very much)
18. If you had the choice, which would you prefer between the two modes of delivery?	o Intramuscolar<br>o Nasal<br>o I don't mind
19 A. In any vaccination, how much would you be willing to vaccinate yourself with intramuscular vaccine on a scale from 1 to 10?<br><br>19 B. In any vaccination, how much would you be willing to vaccinate yourself with nasal spray on a scale from 1 to 10?	1= nothing<br>10=very much
20. Which of the following variables impact more on your attitude toward vaccination? You can select more than one option.	o Type of vaccine (e.g., mRNA, inactivated live virus, etc.).<br>o Mode of administration (needle vs. spray)<br>o Confidence in pharmaceutical companies<br>o Mistrust in pharmaceutical companies<br>o Confidence in health care personnel and physicians<br>o Mistrust in health care personnel and physicians<br>o Confidence in scientific research<br>o Mistrust in scientific research<br>o Opinions of friends or relatives<br>o Mass media, newspapers, magazines<br>o Other: ...
21. I would be more confident with nasal administration of COVID-19 vaccine.	1-4 (1=Not at all; 4=Very much)
22. I have had COVID-19 vaccine.	o YES<br>o NO

Table S2. Parcels' composition

Latent Variables	Parcels	Items
**Intolerance of Uncertainty**	INCA	*(ITEM 1+ ITEM3+ ITEM 4 + ITEM 10) /4*
	INCB	*(ITEM 5+ ITEM 6+ ITEM 9+ ITEM 12) /4*
	INCC	*(ITEM 2+ ITEM 7+ ITEM 8 + ITEM 11) / 4*
**Persecutory Ideation**	PAA	*(ITEM 1+ ITEM 2+ ITEM 3+ (ITEM 4+ ITEM 5) / 5*
	PAB	*ITEM 6 + ITEM 7+ ITEM 8+ ITEM 9 + ITEM 10) / 5*
**Perceived control**	COA	*(ITEM 1 + ITEM 2R + ITEM 5R+ ITEM 7R+ ITEM 8R) / 5*
	COB	*(ITEM 12+ ITEM 24R+ ITEM 14R + ITEM 16R + ITEM 29) /5*
	COC	*(ITEM 15R+ITEM 20R+ITEM 19 +ITEM 25R + ITEM 23R) / 5*
**Vaccine Hesitancy**	VAXC	*(ITEM 7 + ITEM 9) / 2*
	VAXD	*(ITEM 3 + ITEM 4 + ITEM 6) / 3*
**Attitude toward intranasal vaccine**	VAXA	*(ITEM 14 + ITEM 16) / 2*
	VAXB	*(ITEM 12 + ITEM 13 + ITEM 15) / 3*

Table S3. Effects of socio-demographic variables on dependent varaibles

**Standardized direct of Covariates on dependent variables**				
Effects- Model 3a	*B*	*S.E.*	*p*	*95% CI*
Age → Attitude	-.001	.004	.86	[-.006,.009]
Gender → Attitude	-.036	.061	.56	[-.156,.091
Education → Attitude	.001	.034	.99	[-.062,.074]
Healthcare Worker → Attitude	-.046	.067	.49	[-.167,.098]
Organic Disease → Attitude	.091	.057	.10	[-.001,.226]
Age → Hesitancy	.001	.003	.85	[-.005,.006]
Gender → Hesitancy	-.126*	.056	.03	[-.023, .244]
Education → Hesitancy	-.029	.032	.37	[-.032,.094]
Healthcare Worker → Hesitancy	-.213**	.066	.00	[-.090,-.342]
Organic Disease → Hesitancy	.003	.049	.95	[-.096,.093]
Age →Control	.007	.002	.00	[.010,.003]
Gender → Control	-.032	.067	.63	[-.095,.168]
Education → Control	.014	.034	.68	[-.080,.051]
Healthcare Worker → Control	-.094	.058	.11	[-.211,.019]
Organic Disease → Control	.002	.063	.97	[-.113,.133]
